# Risk factors for venous thromboembolism in patients with chronic kidney disease: a systematic review and meta-analysis

**DOI:** 10.1080/0886022X.2024.2431149

**Published:** 2024-11-25

**Authors:** Ya Zhan, Xinwei Fu, Weihong Bi, Guisen Li

**Affiliations:** a Renal Department, The Third Hospital of Mianyang, Sichuan Mental Health Center, Mianyang, China; b School of Medicine, Renal Department and Nephrology Institute, Sichuan Provincial People’s Hospital, University of Electronic Science and Technology of China, Chengdu, China

**Keywords:** Venous thromboembolism, deep venous thrombosis, pulmonary embolism, chronic kidney disease, meta-analysis

## Abstract

**Purpose:**

The objective of this study was to conduct a meta-analysis and systematic review to identify the risk factors contributing to thromboembolism (VTE) in chronic kidney disease (CKD) patients.

**Methods:**

PubMed, Cochrane Library, Web of Science, and Embase databases were searched from inception to October 2024. Cohort, case-control, and cross-sectional studies examining risk factors for VTE in CKD were included. Quality assessment and data extraction were conducted.

**Results:**

Fourteen studies were analyzed. CKD stage 2 (OR: 1.15, 95% CI: 1.06–1.25, *p* < .01), CKD stage 3 (OR: 2.28, 95% CI: 1.76–2.95, *p* < .01), CKD stage 3–4 (OR: 1.57, 95% CI: 1.17–2.1, *p* < .01), end-stage renal disease (OR: 3.68, 95% CI: 1.02–13.27, *p* < .01), female (OR: 1.30, 95% CI: 1.15–1.48, *p* < .01), congestive heart failure (CHF) (OR: 1.26, 95% CI: 1.09–1.46, *p* < .01), atrial fibrillation (AF) (OR: 1.97, 95% CI: 1.40–2.97, *p* < .01), coronary artery disease (CAD) (OR: 1.28, 95% CI: 1.05–1.56, *p* = .01), and systemic lupus erythematosus (SLE) (OR: 3.06, 95% CI: 1.57–5.94, *p* < .01) were associated with increased risk of VTE. In addition, in sensitivity analyses, hemodialysis increased the risk of VTE compared with peritoneal dialysis (OR: 2.35, 95% CI: 1.34–4.14, *p* < .01).

**Conclusions:**

Different CKD stages, female, CHF, AF, CAD, and SLE emerge as significant risk factors for VTE in CKD patients. Additionally, VTE risk can be influenced by dialysis modality and other factors. Physicians should comprehensively assess the risk of VTE in patients with CKD.

## Introduction

Venous thromboembolism (VTE), which includes deep venous thrombosis (DVT) and pulmonary embolism (PE), is a leading cause of morbidity and mortality worldwide [1]. The incidence rate of VTE varies significantly across different geographical regions. In North American countries like the United States, it stands at approximately 1.1 cases per 1000 person-years. In European countries such as the United Kingdom, the incidence rate is higher, around 1.67 cases per 1000 person-years. In contrast, the incidence in Asian countries is relatively lower, approximately 0.13 cases per 1000 person-years [2]. The incidence of VTE rises exponentially with age and is often accompanied by a high recurrence rate. Studies indicate that approximately 30% of patients experience a recurrence within 10 years [3,4]. VTE places a substantial economic burden on both patients and society, with the annual medical costs for VTE-related hospitalizations and complications estimated at €152.2 billion in Europe and $71 billion in the United States [5,6].

Chronic kidney disease (CKD) is characterized by persistent abnormalities in kidney structure or function, impacting overall health for three months or more. It affects approximately 9.1% of the global population [7]. According to the latest research, the total prevalence of CKD is projected to increase to 436.6 million cases by 2027, representing a 5.8% rise from 2022 [8]. Compared to the general population, individuals with CKD are at significantly higher risk of cardiovascular events, including coronary heart disease, stroke, and heart failure, as well as increased rates of hospitalization and mortality [9,10]. This risk escalates as the estimated glomerular filtration rate (eGFR) declines [11]. Studies have shown that when eGFR falls below 15 mL/min/1.73 m^2^, the risk of cardiovascular events increases 3.4-fold, hospitalization 3.1-fold, and the risk of death 5.9-fold [12]. VTE is a significant complication in patients with CKD. Multiple studies have demonstrated that individuals with CKD have a significantly higher risk of VTE compared to the general population. In individuals with mildly reduced renal function and those with stage 3 or 4 CKD, the incidence of VTE is reported as 1.9 and 4.5 per 1000 person-years, respectively [9,13]. Furthermore, the relative risk (RR) of VTE in these populations is estimated to be 1.28-fold and 2.09-fold higher than that in the general population [14]. Tveit et al. reported a significantly higher incidence of PE among CKD patients requiring dialysis in their cohort study involving 76,718 individuals with end-stage renal disease (ESRD) [15]. Another study demonstrated a 134% increased risk of VTE among patients undergoing dialysis [16]. VTE not only poses a serious health risk but also imposes a financial burden on patients with CKD. Research indicates that the general population has a high mortality rate following VTE, with CKD patients experiencing even greater risks. Specifically, the 30-day mortality rate in CKD patients is 2.56-fold higher compared to non-CKD controls [1,17]. Wattanakit et al. observed that VTE significantly increases the overall disease burden in CKD patients, including elevated treatment costs and decreased productivity [13].

Several studies have identified various risk factors for VTE in patients with CKD, including CKD stage, prolonged bed rest, and the presence of tumors. Variations in study populations, sample sizes, and comorbid conditions contribute to discrepancies in the analysis of VTE risk factors among CKD patients. Currently, no systematic review or meta-analysis has comprehensively assessed the factors influencing VTE occurrence in CKD patients. This study aims to conduct a meta-analysis and systematic review to identify and summarize the risk factors for VTE in CKD patients. This analysis seeks to provide a foundation for improving the clinical effectiveness of VTE prevention and management strategies, ultimately reducing its incidence among CKD patients.

## Materials and methods

### Data sources, search strategy, and selection criteria

This meta-analysis was conducted and reported according to The Priority Reporting Entries for Systematic Evaluation and Meta-Analysis [18] and had been registered in the Prospero system (CRD42024522892).

Comprehensive searches were conducted in the PubMed, Embase, Cochrane Library, and Web of Science databases from their inception through October 2024. Searches were performed using a combination of MeSH terms and free-text keywords. The search terms included ‘chronic kidney disease,’ ‘chronic renal failure,’ ‘CKD,’ ‘CRF,’ ‘venous thrombosis,’ ‘venous thromboembolism,’ ‘deep vein thrombosis,’ ‘pulmonary embolism,’ ‘risk factor,’ and ‘risk score.’ The complete search strategy is listed in Supplementary Table 1. Additionally, investigators manually reviewed references from relevant studies and reviews to ensure comprehensive coverage.

*Inclusion criteria*: (a) Studies included individuals aged 18 years and older with CKD, including those receiving hemodialysis (HD) or peritoneal dialysis (PD). (b) Eligible study designs included case-control, cohort, and cross-sectional studies. (c) Odds ratios (ORs), RR, hazard ratio (HR), and their 95% confidence interval (CI) were directly available in the study. (d) Each included study was independent.

*Exclusion criteria*: (a) The population consisted of non-CKD patients. (b) Individuals younger than 18 years. (c) Animal studies. (d) Duplicate studies or datasets. (e) Data are incomplete or cannot be extracted. (f) Literature types are abstracts, conference papers, case studies, and reviews. Additionally, patients who underwent kidney transplantation were excluded from this study.

Based on KDIGO guidelines, CKD is defined as abnormalities in kidney structure or function persisting for over three months. These abnormalities may be evidenced by hematuria, proteinuria, structural abnormalities detected through imaging or laboratory tests, or a glomerular filtration rate (GFR) of less than 60 mL/min/1.73 m^2^ for over three months [19]. The population included in this study consisted mainly of patients identified as having CKD in the original literature. CKD was categorized based on eGFR as follows: CKD stage 1 (GFR ≥90 mL/min/1.73 m^2^), CKD stage 2 (GFR 60–89 mL/min/1.73 m^2^), CKD stage 3a (GFR 45–59 mL/min/1.73 m^2^), CKD stage 3b (GFR 30–44 mL/min/1.73 m^2^), CKD stage 4 (GFR 15–29 mL/min/1.73 m^2^), and CKD stage 5 (GFR <15 mL/min/1.73 m^2^) [19].

### Data collection and quality assessment

Two researchers (Yz and Xwf) independently screened the literature and extracted relevant data. Disagreements were resolved through discussion and consensus. The collected data included the first author’s name, year of publication, country, study design, mean age of participants, gender distribution, sample size, influencing factors, OR, RR, HR, 95% CI, and whether confounders were adjusted for in the multivariable analysis.

The quality of the studies included was assessed using the Newcastle-Ottawa Scale (NOS) and the Agency for Healthcare Research and Quality (AHRQ) scale. The NOS scale, which totals 9 points, classifies studies into low (0–3 points), moderate (4–6 points), and high quality (7–9 points) categories. The AHRQ scale ranges from 0 to 11 points, with scores of 8–11 indicating high quality, 4–7 indicating moderate quality, and 0–3 indicating low quality.

### Statistical analysis

Statistical analyses were performed using R software ­(version 4.3.1) (R Foundation for Statistical Computing, Vienna, Austria). Heterogeneity across studies was assessed using the *I*^2^ statistic. A fixed effects model was applied if *I*^2^ ≤50% and *p* ≥ .1; otherwise, a random effects model was used. *p* < .05 was considered statistically significant. When more than 10 studies are included, a funnel plot is used to assess publication bias. Sensitivity analyses were conducted by sequentially excluding individual studies to identify sources of heterogeneity and assess the stability of the results.

## Results

### Literature search

**Figure 1.**
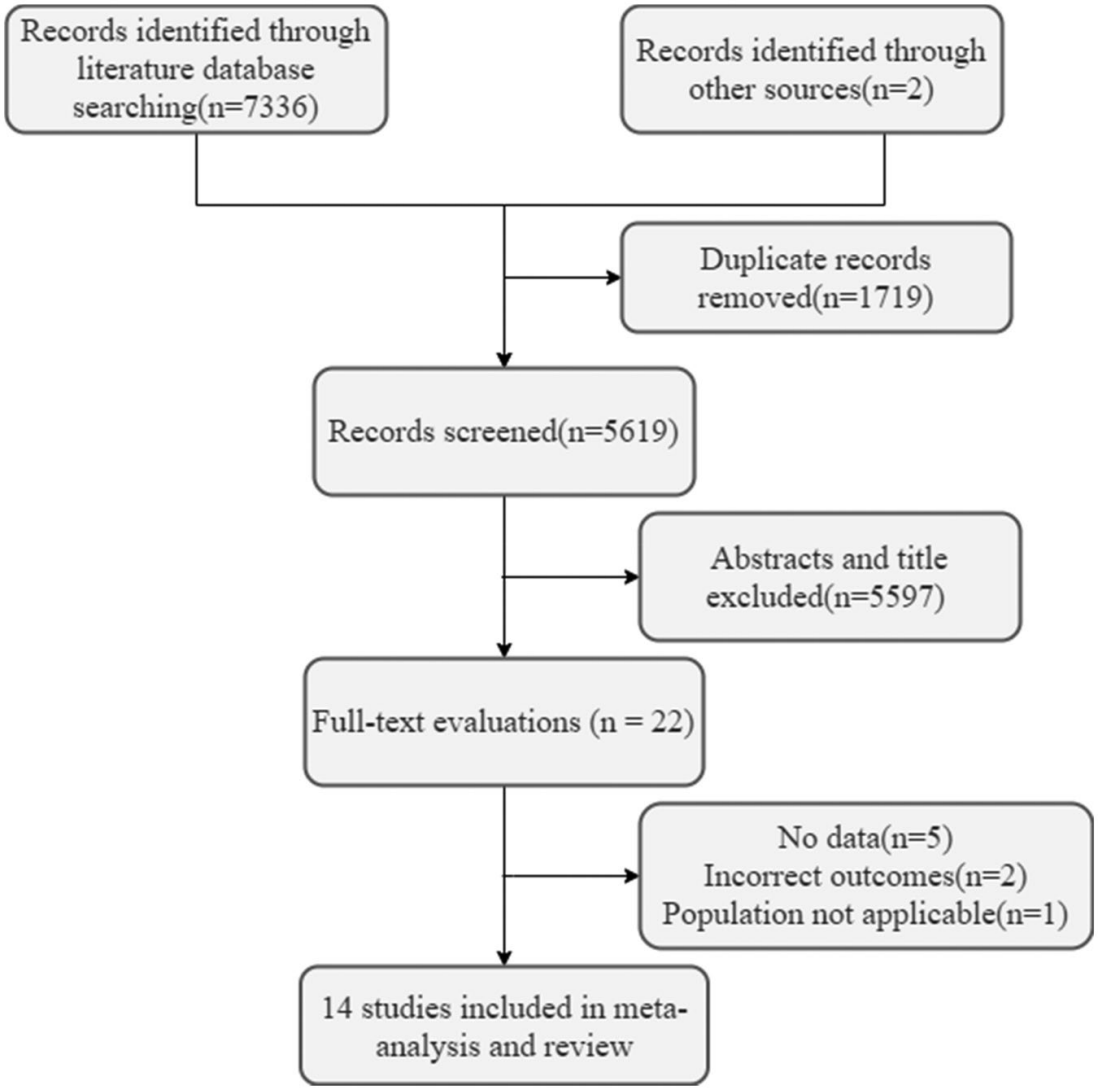
Flow diagram of study selection.

Fourteen studies [13,16,17,20–30] were included in the analysis following a literature search, with the specific screening process outlined in Figure 1. These studies included 1,619,782 participants, of whom 151,469 (9.4%) had VTE. Thirteen studies were classified as high quality, while the remaining one was deemed moderate quality. Among the included studies, five comprised a test and a control group. Of these, three studies included a test group of CKD patients with VTE (VTE group) and a control group of CKD patients without VTE (non-VTE group). Another study included a test group of patients with ESRD and a control group of non-ESRD patients. The final study involved CKD patients receiving erythropoiesis-stimulating agents (ESA group) as the test group, while the control group included CKD patients not receiving ESA treatment (non-ESA group). The characteristics of the 14 included studies are presented in Table 1. A detailed analysis of the quality assessment of the studies can be found in Supplementary data, Tables S2–S4.

**Table 1.** Baseline characteristic of included studies.

Study	Country	Study design	Population	Sample size	Age (years)	VTE (*n*)	Reported factors
Zhang et al. [20]	UK	Cohort	CKD	397,658	56.58^a^	17,890	KDIGO risk, validated polygenic risk score
Cheung et al. [21]	United States	Case-cohort	CKD	1233	VTE group: 69^a^ No VTE group: 68^a^	294	eGFR, statin use, warfarin use, physical activity, BMI
Lu and Liao [22]	China	Cohort	ESRD	ESRD group: 3564 Non-ESRD group: 5094	ESRD group: 62.76^a^ Non-ESRD group: 61.84^a^	226	ESRD, age, HBP, DM, hyperlipidemia, CHF, AF, surgery, cancer
Cheung et al. [23]	United States	Cohort	CKD	25,102	NA	239	CKD stage, ACR
Königsbrügge et al. [24]	Austria	Cross-sectional	Dialysis	626	66^a^	61	Female, age, DM, AF, stroke, CAD, cancer, smoking
Wang et al. [17]	China	Cohort	Dialysis	14,680	NA	413	ESRD, age, gender, CAD, DM, stroke, hyperlipidemia, AF, HBP, CHF, cancer, SLE, warfarin use, central venous catheter, HD
Christiansen et al. [16]	Denmark	Case-control	CKD	VTE group: 128,096 Control group: 642,426	NA	128,096	Primary kidney diseases, dialysis
Suttorp et al. [25]	Dutch	Cohort	Dialysis	EPO group: 630 No EPO group: 125	ESA group: 61.2^a^ No ESA group: 56.5^a^	13	EPO use
Ocak et al. [26]	Netherlands	Case-control	CKD	VTE group: 2473 Control group: 2936	VTE group: 49.1^a^ Control group: 49.8^a^	2473	CKD stage, surgery, malignancy, immobilization, FV Leiden mutation, prothrombin G20210A
Ocak et al. [27]	Netherlands	Cohort	Dialysis	455	60.4^a^	15	Age, gender, HD, DN, glomerulonephritis, BMI, DM, malignancy, smoking, EPO use, Hb, eGFR, proteinuria, albumin, cholesterol, triglycerides
Folsom et al. [28]	United States	Cohort	CKD	10,700	53–75	228	CKD stage
Ocak et al. [29]	Netherlands	Cohort	CKD	1590	59^a^	49	CKD stage, proteinuria
Wattanakit et al. [13]	United States	Cohort	CKD	19,071	59^a^	413	CKD stage
Tveit et al. [30]	United States	Cohort	Dialysis	363,323	NA	1059	Gender, race, DM, PKD, SLE, PD, CHF

CKD: chronic kidney disease; ESRD: end-stage renal disease; VTE: venous thromboembolism; EPO: erythropoietin; ACR: albumin-to-creatinine ratio; CAD: coronary artery disease; AF: atrial fibrillation; HBP: hypertension; DM: diabetes mellitus; SLE: systemic lupus erythematosus; HD: hemodialysis; PD: peritoneal dialysis; CHF: congestive heart failure; eGFR: estimated glomerular filtration rate; BMI: body mass index; DN: diabetic nephropathy; PKD: polycystic kidney disease; Hb: hemoglobin; UK: United Kingdom.

^a^ Mean.

### CKD stage

**Figure 2.**
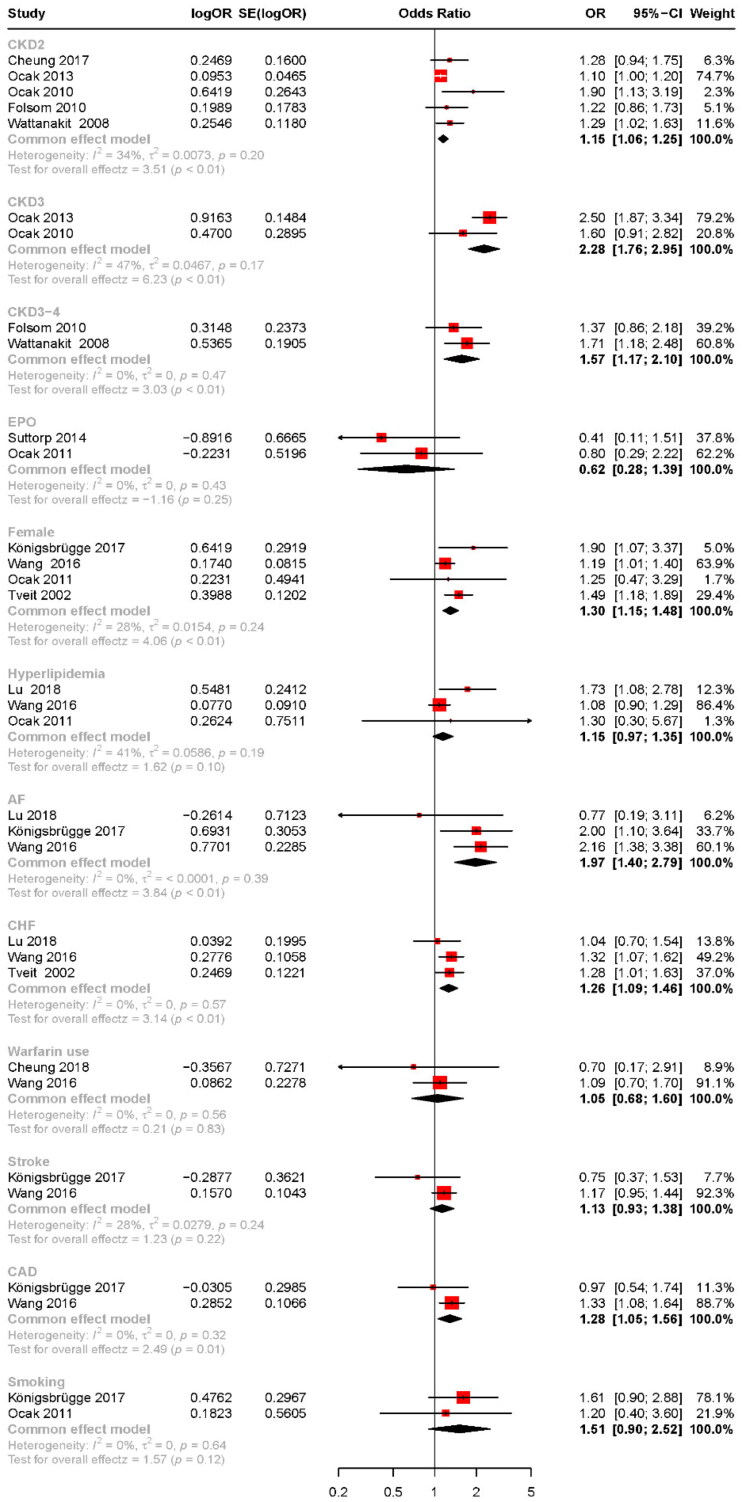
Risk factors for VTE in patients with CKD summarized according to a fixed-effects model. VTE: venous thromboembolism; CKD: chronic kidney disease; EPO: erythropoietin; AF: atrial fibrillation; CHF: congestive heart failure; CAD: coronary artery disease.

**Figure 3.**
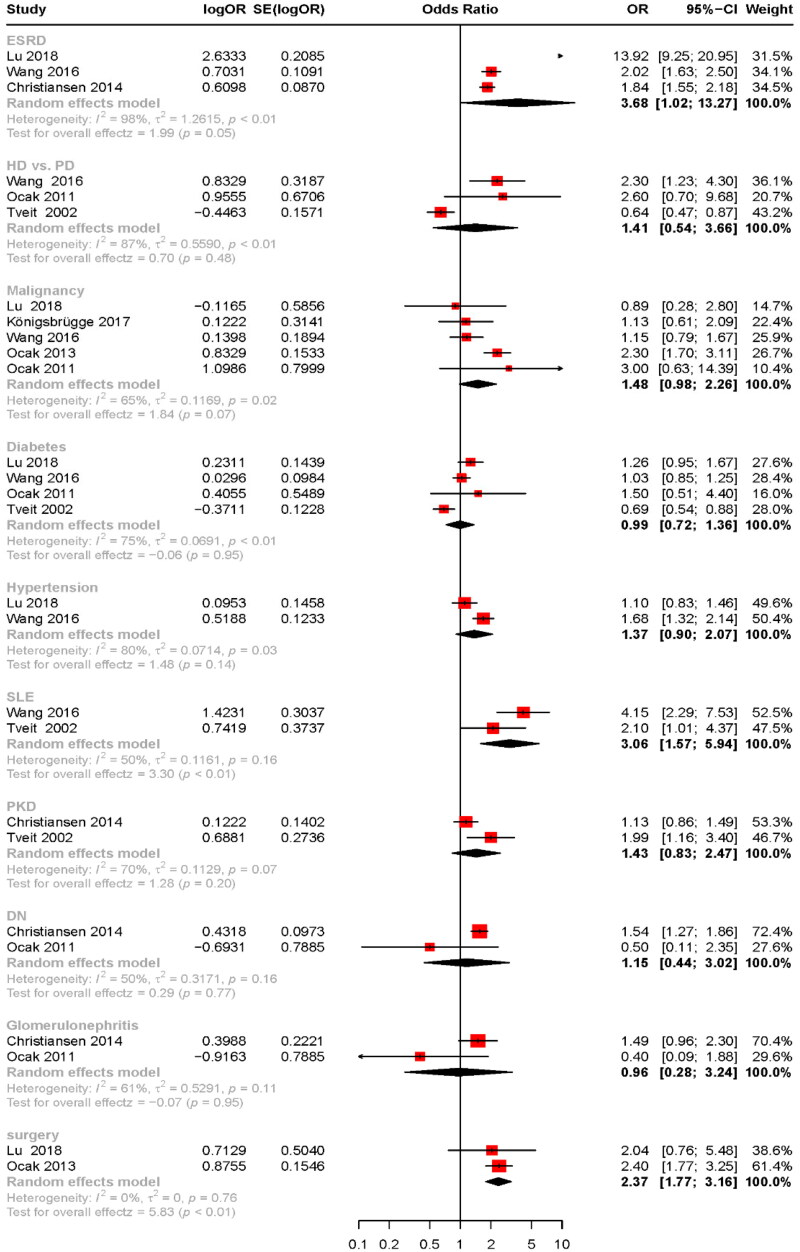
Risk factors for VTE in patients with CKD summarized according to a random-effects model. VTE: venous thromboembolism; CKD: chronic kidney disease; ESRD: end-stage renal disease; HD: hemodialysis; PD: peritoneal dialysis; DM: diabetes mellitus; SLE: systemic lupus erythematosus; DN: diabetic nephropathy.

As shown in Figures 2 and 3, the stages of CKD were found to be associated with an increased risk of VTE. The results are as follows: CKD stage 2 (OR: 1.15, 95% CI: 1.06–1.25, *p* < .01), CKD stage 3 (OR: 2.28, 95% CI: 1.76–2.95, *p* < .01), CKD stage 3–4 (OR: 1.57, 95% CI: 1.17–2.10, *p* < .01), and ESRD (OR: 3.68, 95% CI: 1.02–13.27). The study by Cheung et al. reported an increased risk of VTE associated with CKD stage 3a and CKD stage 3b and below, with ORs of 1.30 (95% CI: 0.7–2.18) for CKD3a and 2.13 (95% CI: 1.21–3.76) for CKD3b and below, respectively [23]. Ocak et al. reported a 5.5-fold increase in the risk of VTE for patients with CKD stages 4–5 [26]. Cheung et al. demonstrated that for every 10 mL/min/1.73 m^2^ reduction in eGFR, the risk of VTE increased by 1.13-fold [21]. Zhang et al. showed that patients classified as intermediate, high, and very high risk according to KDIGO guidelines exhibited a 1.278-fold and a 1.892-fold increase in VTE risk, respectively, compared to those at low risk. Similarly, PE’s risks increased by 1.301-fold and 1.996-fold, respectively [20].

### Primary kidney disease

As shown in Figure 3, the results indicated no significant correlation between incident VTE and PKD, DN, or glomerulonephritis, with ORs and 95% CIs as follows: PKD, OR: 1.43 (95% CI: 0.83–2.47, *p* = .2); DN, OR: 1.15 (95% CI: 0.44–3.02, *p* = .77); glomerulonephritis, OR: 0.96 (95% CI: 0.28–3.24, *p* = .95). Systemic lupus erythematosus (SLE) increases the risk of VTE in patients with CKD (OR: 3.06, 95% CI: 1.57–5.94, *p* < .01). Christiansen et al. reported that patients with CKD whose primary diseases were hypertensive nephropathy, chronic pyelonephritis, and nephrotic syndrome experienced a 1.26-fold, 1.47-fold, and 1.70-fold increased risk of VTE, respectively [16].

### Comorbidity

As shown in Figure 2, significant associations with the occurrence of VTE were observed in CKD patients for congestive heart failure (CHF) (OR: 1.26, 95% CI: 1.09–1.46, *p* < .01), atrial fibrillation (AF) (OR: 1.97, 95% CI: 1.40–2.97, *p* < .01), and coronary artery disease (CAD) (OR: 1.28, 95% CI: 1.05–1.56, *p* = .01).

### Proteinuria

Christiansen et al. demonstrated that compared to an albumin-to-creatinine ratio (ACR) of less than 10 mg/g, the HRs for VTE were 1.14 (95% CI: 0.84–1.55) for an ACR between 10 and 30 mg/g, 1.15 (95% CI: 0.79–1.68) for an ACR between 30 and 300 mg/g, and 0.65 (95% CI: 0.26–1.64) for an ACR of 300 mg/g or higher, indicating no significant association between proteinuria and VTE [16]. In the 2011 study by Ocak et al. [27], it was observed that, compared with proteinuria levels of 0–0.3 g/d, the HRs for proteinuria levels between 0.3 and 3.5 g/d and for levels of 3.5 g/d or higher were 0.3 (95% CI: 0.1–1.1) and 0.8 (95% CI: 0.2–3.2), respectively. However, in a 2010 study by Ocak et al. it was reported that CKD patients with proteinuria-positive urine (urine protein ≥30 mg/d) had a significantly higher risk of VTE compared to those with proteinuria-negative urine (urine protein <30 mg/d), with an HR of 2.1 (95% CI: 1.4–3.2) [29].

### Age and gender

Compared to males, females have an increased risk of VTE (Figure 2). Four studies reported the relationship between age and VTE, two of which demonstrated a significant association. Wang et al. found that the risk of PE increases with age, reporting HRs of 1.47 for patients aged 50–64 years and 2.17 for those aged 65 years and older, compared to patients aged 20–49 years. Similarly, Lu and Liao reported that the risk of VTE is elevated in patients over the age of 50 compared to those under 50 [22]. However, Königsbrügge et al. found no significant relationship between age and the occurrence of VTE [24].

### Hemoglobin and albumin

Patients with hemoglobin levels between 10.47 and 11.60 g/dL and greater than 11.60 g/dL did not exhibit a significantly increased risk of VTE compared to those with hemoglobin levels below 10.47 g/dL. Similarly, patients with albumin levels between 30.1 and 35.5 g/L and greater than 35.5 g/L did not have a significantly increased risk of VTE compared to those with albumin levels below 30.1 g/L [27].

### Physical activity

CKD patients with immobilization face a significantly increased risk of VTE, with an OR of 17.1 (95% CI: 6.8–43.0) [26]. The 2018 study by Cheung et al. found that engaging in physical activity 1–3 times per week and more than four times per week did not significantly affect the risk of VTE compared to patients who did not engage in physical activity [21].

### Body mass index

No correlation between body mass index (BMI) and VTE in CKD patients was found in two studies. Ocak et al. found that patients with a BMI ≥30 kg/m^2^ were not associated with an increased risk of VTE compared to those with a BMI <30 kg/m^2^ (HR: 1.6, 95% CI: 0.4–5.8) [27]. Similarly, the study by Cheung et al. found that CKD patients with BMI <25 kg/m^2^ (HR: 1.07, 95% CI: 0.51–2.22) were not associated with the occurrence of VTE events compared to those with BMI ≥25 kg/m^2^ [21].

### Gene

Factor V Leiden mutation and the prothrombin G20210A mutation increased the risk of VTE [26]. Zhang et al. demonstrated an increased risk of VTE in patients at high genetic risk compared to those at low genetic risk [20].

### Other factors

Tveit et al. found no significant increase in the risk of PE in African American patients and Asian American patients compared to other races [30]. Wang et al. found that central venous catheter use increased the risk of PE in patients with HD [17].

### Publication bias and sensitivity analysis

When more than 10 studies are included, a funnel plot is used to assess publication bias. However, due to the limited number of publications included in this study for each risk factor, publication bias detection could not be performed. Therefore, we conducted a sensitivity analysis.

**Figure 4.**
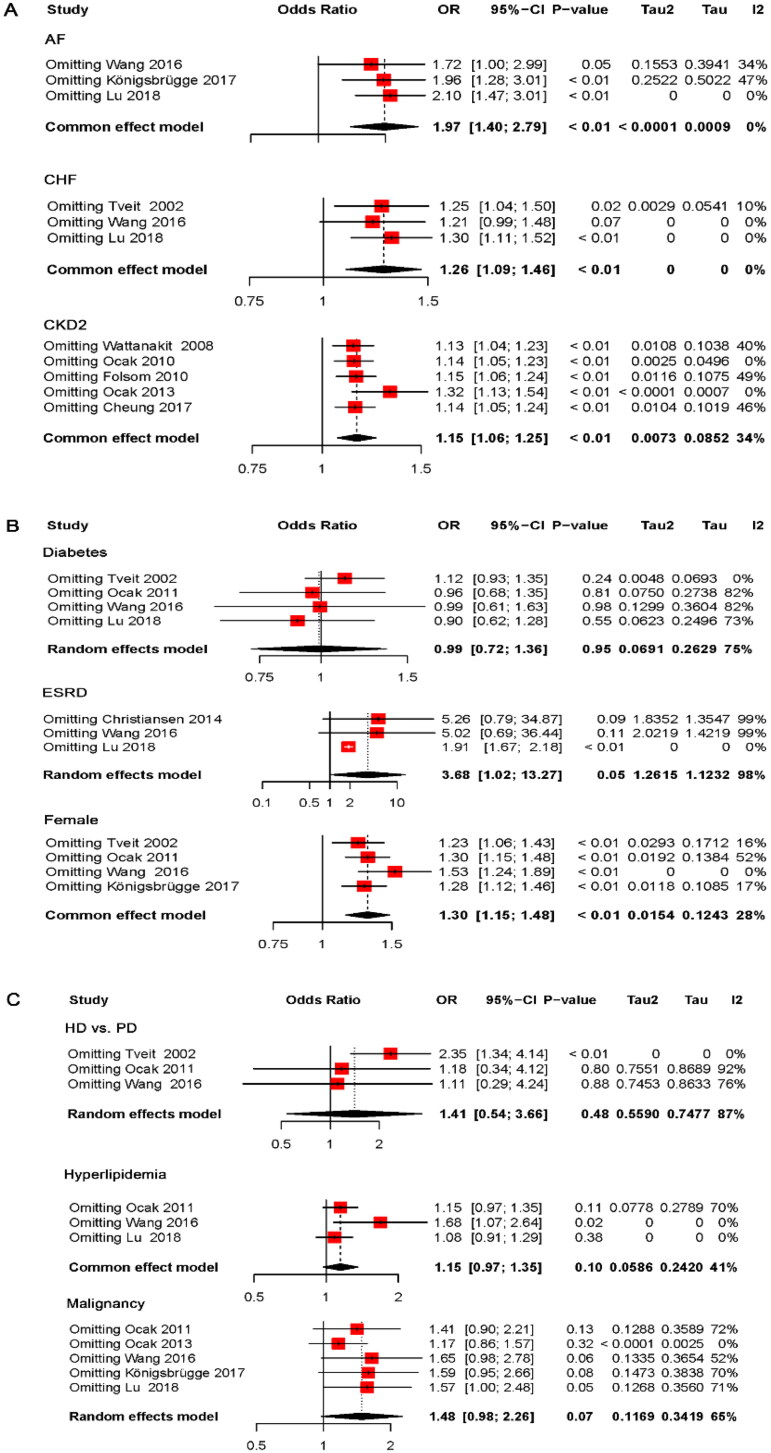
Sensitivity analysis of risk factors for VTE in patients with CKD. VTE: venous thromboembolism; CKD, chronic kidney disease; AF: atrial fibrillation; CHF: congestive heart failure; ESRD: end-stage renal disease; HD: hemodialysis; PD: peritoneal dialysis.

As shown in Figure 4, sensitivity analyses were conducted by individually removing studies from the analysis. The heterogeneity for CKD stage 2, female gender, AF, and CHF did not change significantly, and the results remained stable. The heterogeneity for DM was reduced after the study by Tveit et al. was excluded. An analysis using a fixed-effects model indicated that DM did not increase the risk of VTE (OR: 1.12, 95% CI: 0.93–1.35). HD was found to increase the risk of VTE compared to PD after excluding the study by Tveit et al. (OR: 2.35, 95% CI: 1.34–4.14). The heterogeneity associated with malignancy decreased after excluding the study by Ocak et al. [26]. Even with a fixed-effects model, the analysis suggested that malignancy did not increase the risk of VTE (OR: 1.17, 95% CI: 0.86–1.57).

## Discussion

Multiple factors influence VTE. We conducted the first systematic review and meta-analysis to identify and evaluate all reported risk factors for VTE in patients with CKD, which provides an essential basis for clinical prevention and reduction of VTE occurrence. This study confirmed that VTE in patients with CKD is influenced by various factors, including different stages of CKD, female, CHF, AF, CAD, and SLE. Additionally, factors such as dialysis modality, genetics, central venous catheter use, and age also play significant roles.

Patients with CKD are at an elevated risk for VTE, and this risk escalates with the progression of CKD stages. This study reveals an increased risk of VTE, with patients at CKD stage 2, stage 3–4, stage 3, and ESRD experiencing a 1.15, 1.57, 2.28, and 3.68 times higher risk, respectively. Additionally, another study reported a 1.13-fold increase in the risk of VTE for every 10 mL/min/1.73 m^2^ decrease in eGFR [21]. CKD staging is strongly associated with the risk of VTE, and the potential mechanisms involved can be analyzed as follows: on the one hand, as kidney function declines, the accumulation of metabolic waste products increases, leading to elevated blood viscosity, which promotes thrombus formation. Additionally, imbalances in electrolytes such as calcium and potassium can disrupt the coagulation mechanism, further elevating the risk of thrombus formation. On the other hand, the decline in renal function is linked to impaired vascular endothelial function, alterations in levels of crucial coagulation factors such as fibrinogen, and subsequent inflammatory responses, all of which contribute to the increased risk of VTE [31–33]. Several studies have demonstrated a strong link between chronic inflammation and the development of VTE, particularly in patients with CKD [16,21,34].

The primary underlying disease in CKD significantly affects the risk of VTE. This study demonstrated that SLE increased the risk of VTE by 3.06-fold, whereas PKD, DN, and glomerulonephritis did not significantly alter the risk. In another scholarly report, hypertensive nephropathy, chronic pyelonephritis, and nephrotic syndrome were associated with a 1.26-fold, 1.47-fold, and 1.7-fold increased risk of VTE, respectively [16]. The combined results of the final meta-analysis were somewhat influenced by the limited data available in the literature on the primary diseases of CKD included in this study.

It is well established that patients with CKD often present with multiple cardiovascular comorbidities, such as CAD and AF. This study demonstrated that CHF, CAD, and AF all increase the risk of VTE, with CKD patients who have concomitant AF experiencing a 97% higher risk of VTE compared to those without AF. Both CHF and AF involve significant hemodynamic alterations, which play a crucial role in the development of VTE [35,36]. CAD is often accompanied by atherosclerosis and impaired endothelial function, both of which are more pronounced in patients with CKD. CKD patients with comorbid CAD experienced a 28% increased risk of VTE compared to CKD patients without comorbid CAD. These findings suggest that cardiovascular disease in CKD patients significantly increases the risk of VTE. Therefore, comprehensive management and evaluation of these patients is essential to reduce their risk of VTE.

Additionally, this study found that HBP, DM, and hyperlipidemia were not associated with an increased risk of VTE in CKD patients. However, these findings may be influenced by the limited number of studies included. Notably, the survey by Ocak et al. also indicated that HBP and DM do not contribute to the increased risk of VTE in CKD patients [29].

Cancer is a recognized high-risk factor for VTE in the general population [37]. However, after summarizing multiple studies, this research found that cancer was not identified as a risk factor for VTE in patients with CKD. Sensitivity analyses further indicated that cancer was not a risk factor for VTE in CKD patients, suggesting that our statistical results are stable. The potential reasons for this may include the following: first, the sample size of cancer patients in these studies was relatively small, and certain specific types of tumors were underrepresented, potentially reducing the observed impact of cancer on VTE. Second, CKD patients exhibit significant alterations in coagulation and anticoagulation mechanisms due to impaired renal function [38–40], which may obscure or attenuate the influence of other known risk factors, such as cancer. Therefore, although cancer was recognized as a high-risk factor for VTE in the general population, the pathophysiology of VTE in CKD patients is more complex, and further research is needed to clarify the role of cancer in VTE development among CKD patients.

This study identified gender differences in the risk of VTE, with female patients with CKD being more likely to develop VTE compared to their male counterparts. Previous studies have suggested that women may be more susceptible to VTE than men [41], potentially due to factors such as the use of estrogen-containing birth control pills and pregnancy [42]. While a normal BMI and increased physical activity are associated with a lower risk of VTE in the general population [43,44], our systematic review found that BMI and physical activity levels in patients with CKD were not associated with VTE development. In patients without CKD, achieving a normal BMI may reduce the risk of VTE [44]. This systematic review found that some studies suggest an elevated BMI in CKD patients is not associated with an increased risk of VTE. One hypothesis to explain this is that the inflammatory or pro-coagulant pathways in CKD may outweigh any potential effect of a normal BMI in reducing VTE risk in CKD patients. Additionally, a lower BMI in CKD patients may be due to unmeasured confounding illnesses, which could obscure the protective effect of a lower BMI against VTE [21]. However, immobilization was found to increase the risk of VTE in this population significantly.

Genetic factors play a significant role in the development of VTE. In patients with CKD, the factor V Leiden mutation and prothrombin G20210A mutation are associated with a 4.3-fold and 9.5-fold increased risk of VTE, respectively [26]. Since the 1990s, awareness of the genetic influence on VTE risk has grown, particularly with recognizing activated protein C resistance [45]. A study by Zhang et al. suggested that genetic predisposition leads to varying VTE risks among CKD patients [20]. These findings underscore the importance of considering inherited coagulation disorders when managing CKD patients, particularly in risk assessments and the development of prevention strategies.

The type of dialysis influences the risk of VTE. In this study, HD was found to increase the risk of VTE compared to PD through sensitivity analysis. Molnar et al. analyzed 13,315 adult dialysis patients and found that while the risk of VTE existed in PD and HD patients, the incidence was higher in those undergoing HD [46]. This increased risk may be attributed, on the one hand, to the rapid hemodynamic changes in HD patients, which can cause vascular wall damage, and on the other hand, to biocompatibility issues associated with dialysis equipment during HD, potentially activating inflammatory pathways that elevate the risk of thrombus formation.

### Advantages and limitations

This study represents the largest and most comprehensive review and meta-analysis of factors influencing the development of VTE in patients with CKD. To ensure maximal completeness of results, we conducted an extensive literature search to identify potential studies for inclusion. Strict inclusion and exclusion criteria were applied, and studies from multiple countries were incorporated to enhance the rigor of the results and ensure diverse representation.

There are several limitations to this study. First, some articles did not explicitly state the screening methods used for VTE, and variations in screening methods may have influenced the reported prevalence of VTE. Second, there is a potential for publication bias, which may arise from the limited number of studies investigating certain risk factors and the lack of capacity to conduct subgroup analyses to detect bias. Consequently, further validation of the results is necessary. Third, some studies failed to report adjusted estimates, potentially leaving their findings vulnerable to confounding factors. Finally, certain risk factors exhibited high heterogeneity, which may have impacted the overall results.

## Conclusions

Various factors, including different stages of CKD, female, CHF, AF, CAD, and SLE, play significant roles in VTE development among CKD patients. Additionally, VTE is influenced by factors like dialysis modality, genetics, central venous catheterization, and age. Physicians should comprehensively assess patients’ VTE risk and develop targeted strategies to prevent and mitigate this risk. Furthermore, future prospective studies are needed to validate this study’s findings and identify risk factors in patients with specific characteristics.

## Supplementary Material

Figure.docx

Supplementary Table.docx

PRISMA_2020_checklist.docx

## Data Availability

The authors confirm that the data supporting the findings of this study are available within the article.

## Geolocation information

No. 190, East Duan, Jiannan Road, Youxian District, Mianyang, Sichuan, China.
