# Supplementary material for: Risk factors for venous thromboembolism in patients with chronic kidney disease: a systematic review and meta-analysis
Source: Ren Fail. 2024 Nov 25;46(2):2431149. doi: 10.1080/0886022X.2024.2431149 (PMC11590193; doi:10.1080/0886022X.2024.2431149)
Supplement: Figure.docx [file IRNF_A_2431149_SM8051.docx]

| 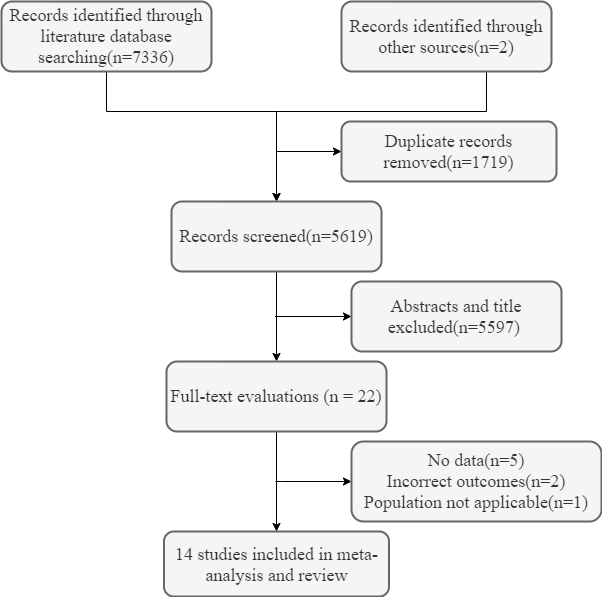 |
| --- |
| Figure 1. Flow diagram of study selection |

| 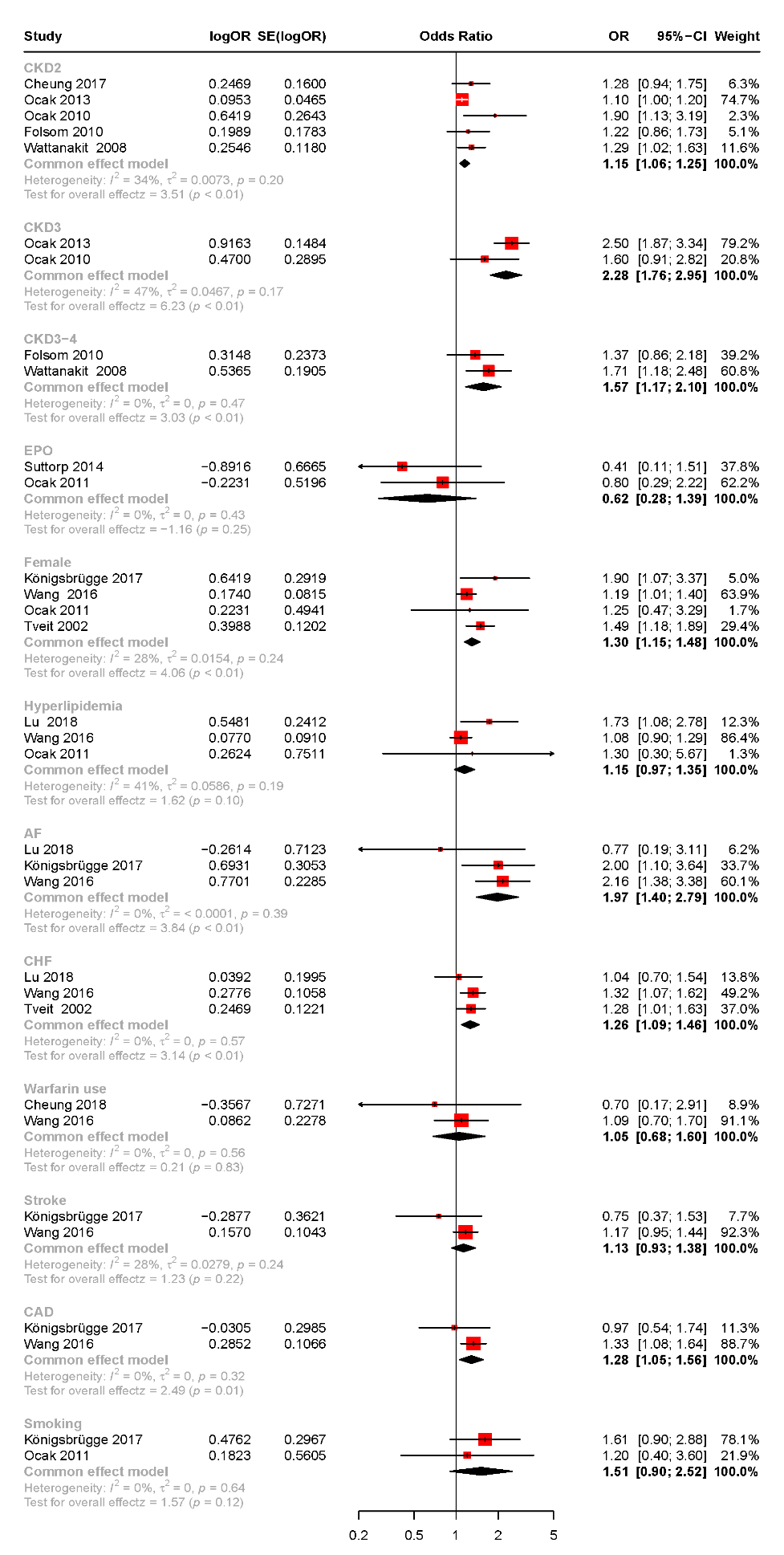 |
| --- |
| Figure 2. Risk factors for VTE in patients with CKD summarized according to a fixed-effects model. VTE, venous thromboembolism; CKD, chronic kidney disease; EPO, erythropoietin; AF, atrial fibrillation; CHF, congestive heart failure; CAD, coronary artery disease |

| 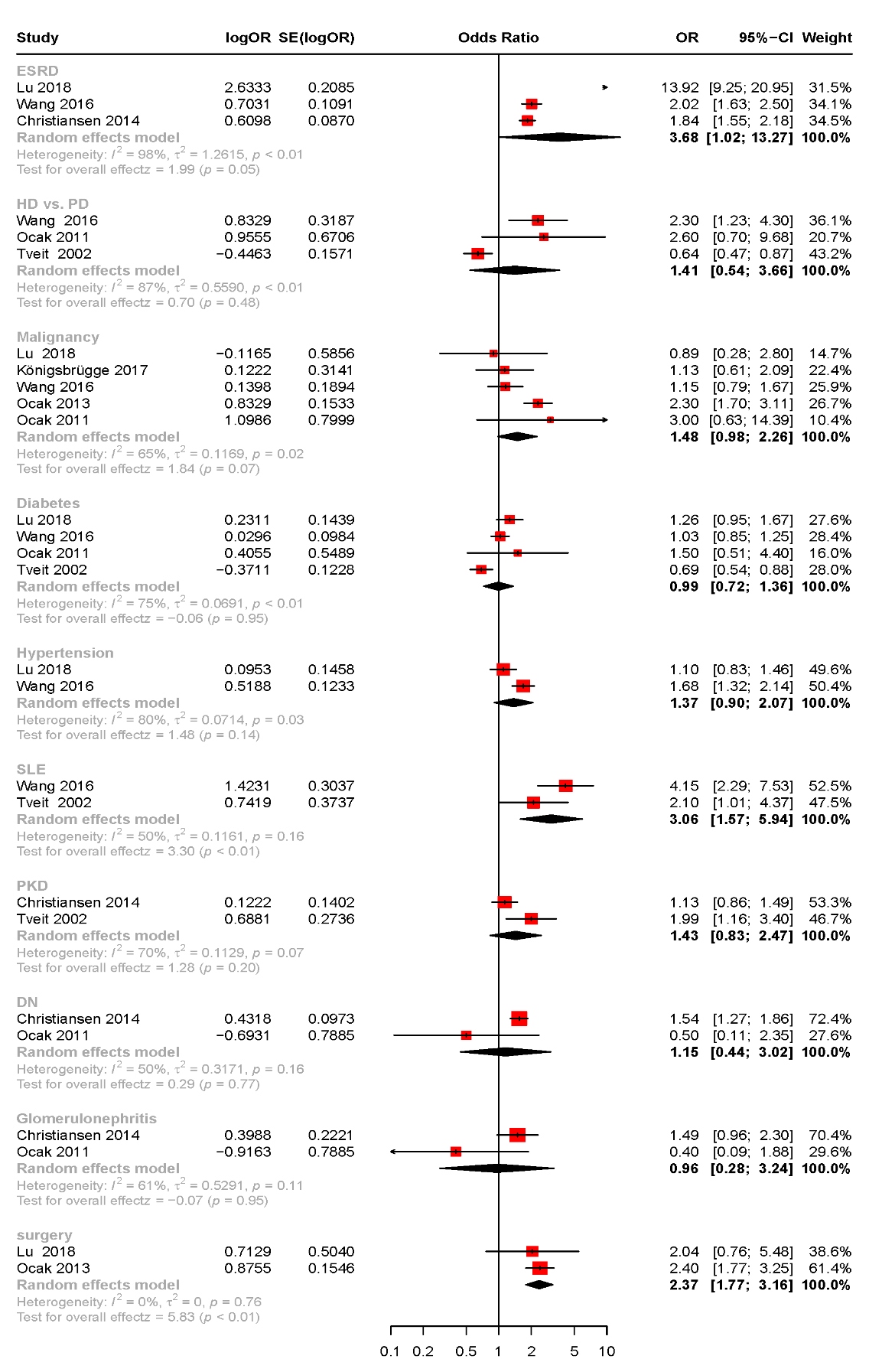 |
| --- |
| Figure 3. Risk factors for VTE in patients with CKD summarized according to a random-effects model. VTE, venous thromboembolism; CKD, chronic kidney disease; ESRD, end-stage renal disease; HD, hemodialysis; PD, peritoneal dialysis; DM, diabetes mellitus; SLE, systemic lupus erythematosus; DN, diabetic nephropathy |

| 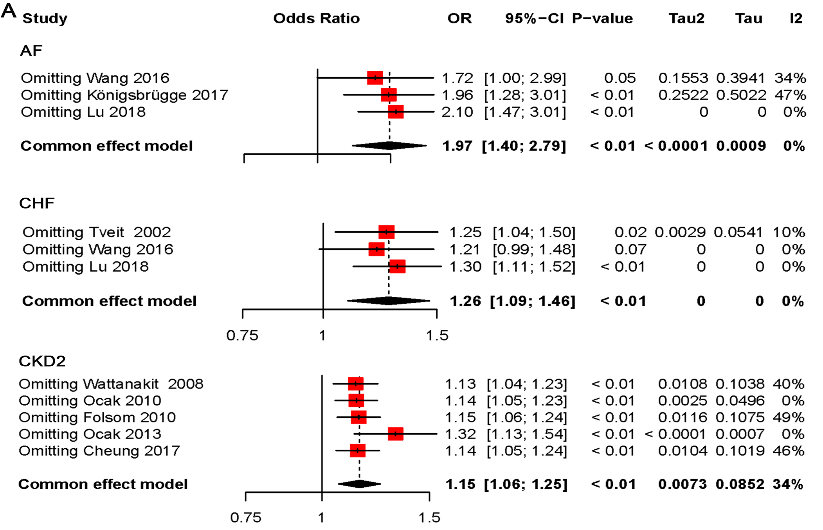  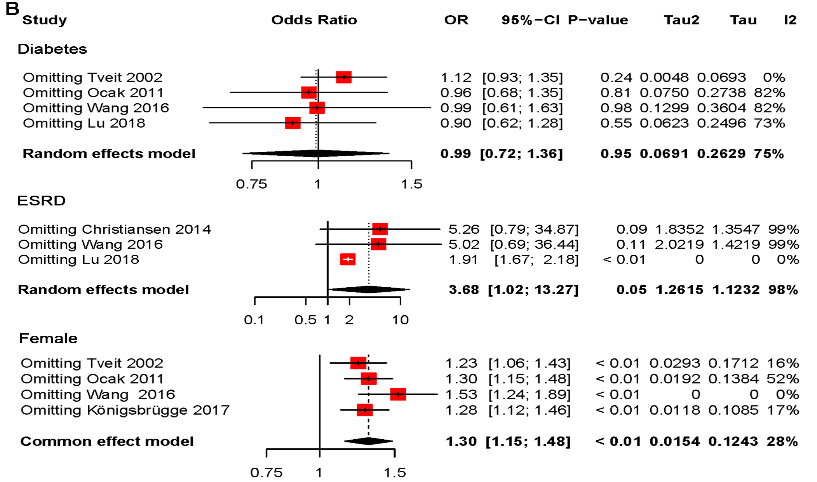  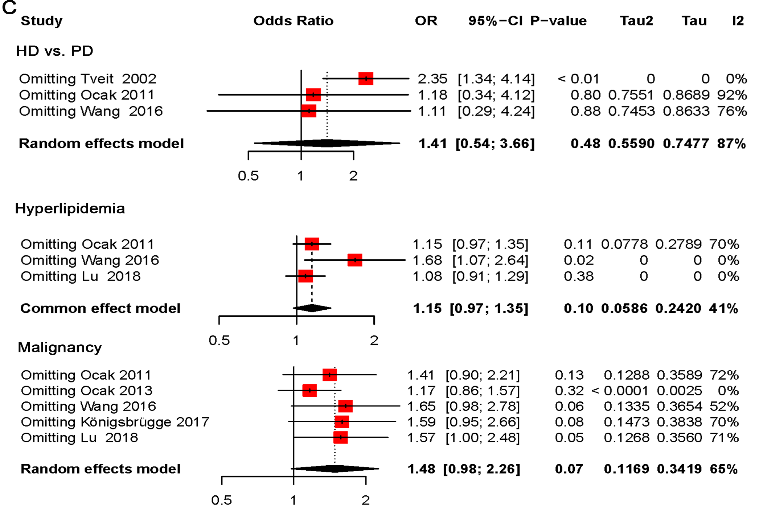 |
| --- |
| Figure 4. Sensitivity analysis of risk factors for VTE in patients with CKD. VTE, venous thromboembolism; CKD, chronic kidney disease; AF, atrial fibrillation; CHF, congestive heart failure; ESRD, end-stage renal disease; HD, hemodialysis; PD, peritoneal dialysis |
