# Supplementary material for: Risk factors for venous thromboembolism in patients with chronic kidney disease: a systematic review and meta-analysis
Source: Ren Fail. 2024 Nov 25;46(2):2431149. doi: 10.1080/0886022X.2024.2431149 (PMC11590193; doi:10.1080/0886022X.2024.2431149)
Supplement: Supplementary Table.docx [file IRNF_A_2431149_SM8050.docx]

| Table of contents | |
| --- | --- |
| Tables | Supplementary Table 1: Complete search strategy |
|  | Supplementary Table 2: Quality assessment of cohort studies |
|  | Supplementary Table 3: Quality assessment of case-control studies |
|  | Supplementary Table 4: Quality assessment of cross-sectional study |

**Supplementary Table 1: Complete search strategy**

1. pubmed

| Search number | Query | Results |
| --- | --- | --- |
| #1 | (((((((((risk factor[MeSH Terms]) OR (Social Risk Factors)) OR (Social Risk Factor)) OR (Health Correlates)) OR (Population at Risk)) OR (Populations at Risk)) OR (Risk Scores)) OR (Risk Score)) OR (Risk Factor Scores)) OR (Risk Factor Score) | 2,240,808 |
| #2 | ((((((((((((((((((((((((((((((((chronic kidney disease[MeSH Terms]) OR (Chronic renal failure)) OR (Uremia)) OR (Renal Dialysis)) OR (Chronic Renal Insufficiencies)) OR (Chronic Renal Insufficiency)) OR (Chronic Kidney Insufficiency)) OR (Chronic Kidney Insufficiencies)) OR (chronic kidney diseases)) OR (Chronic Renal Disease)) OR (Chronic Renal Diseases)) OR (CKD)) OR (CRF)) OR (End-Stage Kidney Disease)) OR (End Stage Kidney Disease)) OR (Chronic Kidney Failure)) OR (End-Stage Renal Disease)) OR (End Stage Renal Disease)) OR (End-Stage Renal Failure)) OR (Chronic Renal Failure)) OR (ESRD)) OR (Uremias)) OR (Renal Dialyses)) OR (Hemodialysis)) OR (Hemodialyses)) OR (Extracorporeal Dialyses)) OR (Extracorporeal Dialysis)) OR (renal replacement therapy)) OR (Renal Replacement Therapies)) OR (Kidney Replacement Therapies)) OR (Kidney Replacement Therapy)) OR (peritoneal dialysis)) OR (Peritoneal Dialyses) | 480,559 |
| #3 | ((((((((((((((((((((((((Venous Thrombosis[MeSH Terms]) OR (Thrombosis)) OR (Thromboembolism)) OR (Venous Thromboembolism)) OR (Pulmonary Embolism)) OR (Phlebothrombosis)) OR (Phlebothromboses)) OR (Venous Thromboses)) OR (Deep Vein Thrombosis)) OR (Deep Vein Thromboses)) OR (Deep-Venous Thrombosis)) OR (Deep-Venous Thromboses)) OR (Deep-Vein Thrombosis)) OR (Deep-Vein Thromboses)) OR (Deep Venous Thrombosis)) OR (Deep Venous Thromboses)) OR (Thromboses)) OR (Thrombus)) OR (Blood Clot)) OR (Blood Clots)) OR (Thromboembolisms)) OR (Thromboembolism)) OR (Pulmonary Embolisms)) OR (Pulmonary Thromboembolisms)) OR (Pulmonary Thromboembolism) | 410,758 |
| #4 | #1 AND #2 AND #3 | 3,192 |

1. Cochrane

| Search number | Query | Results |
| --- | --- | --- |
| #1 | MeSH descriptor: [Renal Insufficiency, Chronic] explode all trees | 9,853 |
| #2 | (Chronic renal failure or Uremia or Renal Dialysis or Chronic Renal Insufficiencies or Chronic Renal Insufficiency or Chronic Kidney Insufficiency or Chronic Kidney Insufficiencies or chronic kidney diseases or Chronic Renal Disease or Chronic Renal Diseases or CKD or CRF or End-Stage Kidney Disease or End Stage Kidney Disease or Chronic Kidney Failure or End-Stage Renal Disease or End Stage Renal Disease or End-Stage Renal Failure or Chronic Renal Failure or ESRD or Uremias or Renal Dialyses or Hemodialysis or Hemodialyses or Extracorporeal Dialyses or Extracorporeal Dialysis or renal replacement therapy or Renal Replacement Therapies or Kidney Replacement Therapies or Kidney Replacement Therapy or peritoneal dialysis or Peritoneal Dialyses):ti,ab,kw (Word variations have been searched) | 45,216 |
| #3 | #1 or #2 | 45,216 |
| #4 | MeSH descriptor: [Venous Thrombosis] explode all trees | 3,602 |
| #5 | (Thrombosis or Thromboembolism or Venous Thromboembolism or Pulmonary Embolism or Phlebothrombosis or Phlebothromboses or Venous Thromboses or Deep Vein Thrombosis or Deep Vein Thromboses or Deep-Venous Thrombosis or Deep-Venous Thromboses or Deep-Vein Thrombosis or Deep-Vein Thromboses or Deep Venous Thrombosis or Deep Venous Thromboses or Thromboses or Thrombus or Blood Clot or Blood Clots or Thromboembolisms or Thromboembolism or Pulmonary Embolisms or Pulmonary Thromboembolisms or Pulmonary Thromboembolism):ti,ab,kw (Word variations have been searched) | 35,229 |
| #6 | #4 or #5 | 35,953 |
| #7 | MeSH descriptor: [Risk Factors] explode all trees | 38,474 |
| #8 | (Social Risk Factors or Social Risk Factor or Health Correlates or Population at Risk or Populations at Risk or Risk Scores or Risk Score or Risk Factor Scores or Risk Factor Scor):ti,ab,kw (Word variations have been searched) | 107,624 |
| #9 | #7 or #8 | 133,023 |
| #10 | #3 and #6 and #9 | 354 |

**3.** Embase

| Search number | Query | Results |
| --- | --- | --- |
| #1 | 'chronic kidney failure'/exp | 364,312 |
| #2 | 'uremia':ab,ti OR 'renal dialysis':ab,ti OR 'chronic renal insufficiencies':ab,ti OR 'chronic renal insufficiency':ab,ti OR 'chronic kidney insufficiency':ab,ti OR 'chronic kidney insufficiencies':ab,ti OR 'chronic kidney diseases':ab,ti OR 'chronic renal disease':ab,ti OR 'chronic renal diseases':ab,ti OR 'ckd':ab,ti OR 'crf':ab,ti OR 'end-stage kidney disease':ab,ti OR 'end stage kidney disease':ab,ti OR 'chronic kidney failure':ab,ti OR 'end-stage renal disease':ab,ti OR 'end stage renal disease':ab,ti OR 'end-stage renal failure':ab,ti OR 'chronic renal failure':ab,ti OR 'esrd':ab,ti OR 'uremias':ab,ti OR 'renal dialyses':ab,ti OR 'hemodialysis':ab,ti OR 'hemodialyses':ab,ti OR 'extracorporeal dialyses':ab,ti OR 'extracorporeal dialysis':ab,ti OR 'renal replacement therapy':ab,ti OR 'renal replacement therapies':ab,ti OR 'kidney replacement therapies':ab,ti OR 'kidney replacement therapy':ab,ti OR 'peritoneal dialysis':ab,ti OR 'peritoneal dialyses':ab,ti | 367,988 |
| #3 | #1 OR #2 | 466,388 |
| #4 | 'vein thrombosis'/exp | 178,615 |
| #5 | 'thrombosis':ab,ti OR 'venous thromboembolism':ab,ti OR 'pulmonary embolism':ab,ti OR 'phlebothrombosis':ab,ti OR 'phlebothromboses':ab,ti OR 'venous thromboses':ab,ti OR 'deep vein thrombosis':ab,ti OR 'deep vein thromboses':ab,ti OR 'deep-venous thrombosis':ab,ti OR 'deep-venous thromboses':ab,ti OR 'deep-vein thrombosis':ab,ti OR 'deep-vein thromboses':ab,ti OR 'deep venous thrombosis':ab,ti OR 'deep venous thromboses':ab,ti OR 'thromboses':ab,ti OR 'thrombus':ab,ti OR 'blood clot':ab,ti OR 'blood clots':ab,ti OR 'thromboembolisms':ab,ti OR 'thromboembolism':ab,ti OR 'pulmonary embolisms':ab,ti OR 'pulmonary thromboembolisms':ab,ti OR 'pulmonary thromboembolism':ab,ti | 411,365 |
| #6 | #4 OR #5 | 481,484 |
| #7 | 'risk factor'/exp | 1,472,425 |
| #8 | 'social risk factors':ab,ti OR 'social risk factor':ab,ti OR 'health correlates':ab,ti OR 'population at risk':ab,ti OR 'populations at risk':ab,ti OR 'risk scores':ab,ti OR 'risk score':ab,ti OR 'risk factor scores':ab,ti OR 'risk factor score':ab,ti | 81,577 |
| #9 | #7 OR #8 | 1,533,219 |
| #10 | #3 AND #6 AND #9 | 2,186 |

**4.**Web of science

| Search number | Query | Results |
| --- | --- | --- |
| #1 | (TS=(risk factor) OR AB=(Social Risk Factors OR Social Risk Factor OR Health Correlates OR Population at Risk OR Populations at Risk OR Risk Scores OR Risk Score OR Risk Factor Scores OR Risk Factor Score) ) AND (TS=(chronic kidney disease) OR AB=(Chronic renal failure OR Uremia OR Renal Dialysis OR Chronic Renal Insufficiencies OR Chronic Renal Insufficiency OR Chronic Kidney Insufficiency OR Chronic Kidney Insufficiencies OR chronic kidney diseases OR Chronic Renal Disease OR Chronic Renal Diseases OR CKD OR CRF OR End-Stage Kidney Disease OR End Stage Kidney Disease OR Chronic Kidney Failure OR End-Stage Renal Disease OR End Stage Renal Disease OR End-Stage Renal Failure OR Chronic Renal Failure OR ESRD OR Uremias OR Renal Dialyses OR Hemodialysis OR Hemodialyses OR Extracorporeal Dialyses OR Extracorporeal Dialysis OR renal replacement therapy OR Renal Replacement Therapies OR Kidney Replacement Therapies OR Kidney Replacement Therapy OR peritoneal dialysis OR Peritoneal Dialyses) ) AND (TS=(Venous Thrombosis) OR AB=(Thrombosis OR Thromboembolism OR Venous Thromboembolism OR Pulmonary Embolism OR Phlebothrombosis OR Phlebothromboses OR Venous Thromboses OR Deep Vein Thrombosis OR Deep Vein Thromboses OR Deep-Venous Thrombosis OR Deep-Venous Thromboses OR Deep-Vein Thrombosis OR Deep-Vein Thromboses OR Deep Venous Thrombosis OR Deep Venous Thromboses OR Thromboses OR Thrombus OR Blood Clot OR Blood Clots OR Thromboembolisms OR Thromboembolism OR Pulmonary Embolisms OR Pulmonary Thromboembolisms OR Pulmonary Thromboembolism) ) | 1604 |

**Supplementary Table 2: Quality assessment of cohort studies**

| Newcastle-Ottawa Scale | Selection | | | | Comparability | Outcome | | |  |
| --- | --- | --- | --- | --- | --- | --- | --- | --- | --- |
|  | Representativeness of the exposed cohort | Selection of the non exposed cohort | Ascertainment of exposure | Demonstration that outcome of interest was not present at start of study | Comparability of cohorts on the basis of the design or analysis | Assessment of outcome | Was follow-up long enough for outcomes to occur | Adequacy of follow up of cohorts | sore |
| Zhang 2023 | 1 | 1 | 1 | 1 | 2 | 1 | 1 | 1 | 9 |
| Lu 2018 | 1 | 1 | 1 | 1 | 1 | 1 | 1 | 0 | 8 |
| Cheung 2017 | 1 | 1 | 1 | 1 | 2 | 1 | 1 | 1 | 9 |
| Wang 2016 | 1 | 1 | 1 | 1 | 2 | 1 | 1 | 1 | 9 |
| Suttorp 2014 | 1 | 1 | 1 | 1 | 2 | 1 | 1 | 0 | 9 |
| Ocak 2011 | 1 | 0 | 1 | 1 | 1 | 1 | 1 | 0 | 7 |
| Ocak 2010 | 1 | 1 | 1 | 1 | 2 | 1 | 1 | 1 | 9 |
| Folsom 2010 | 1 | 1 | 1 | 1 | 0 | 1 | 1 | 0 | 6 |
| Wattanakit 2008 | 1 | 1 | 1 | 1 | 2 | 1 | 1 | 1 | 9 |
| Tveit 2002 | 1 | 1 | 1 | 1 | 2 | 1 | 1 | 1 | 9 |

**Supplementary Table 3: Quality assessment of case-control studies**

| Newcastle-Ottawa | Selection | | | | Comparability | Exposure | | |  |
| --- | --- | --- | --- | --- | --- | --- | --- | --- | --- |
|  | Is the case definition adequate | Representativeness of the cases | Selection of controls | Definition of controls | Comparability of cases and controls on the basis of the design or analysis | Ascertainment of exposure | Same method of ascertainment for cases and controls | Non-Response rate | sore |
| Cheung 2018 | 1 | 1 | 1 | 1 | 2 | 1 | 1 | 1 | 9 |
| Christiansen 2014 | 1 | 1 | 1 | 1 | 1 | 1 | 1 | 0 | 7 |
| Ocak 2013 | 1 | 1 | 1 | 1 | 1 | 0 | 1 | 1 | 7 |

**Supplementary Table 4: Quality assessment of cross-sectional study**

| Agency for Healthcare Research and Quality  assessment | Define the source of information | List inclusion and exclusion criteria for exposed and unexposed subjects(cases and controls) or refer to previous publications | Indicate time period used for identifying patients | Indicate whether or not subjects were consecutive if not population-based | Indicate if evaluators of subjective components of study were masked to other aspects of the participants | Describe any assessments undertaken for quality assurance purposes | Explain any patient exclusions from analysis | Describe how confounding was assessed and/or controlled | If applicable, explain how missing data were handled in the analysis | Summarize patient response rates and completeness of data collection | Clarify what follow-up, if any, was expected and the percentage of patients for which incomplete data or follow-up was obtained | sore |
| --- | --- | --- | --- | --- | --- | --- | --- | --- | --- | --- | --- | --- |
| Königsügge 2017 | 1 | 1 | 1 | 1 | 1 | 1 | 1 | 0 | 0 | 1 | 0 | 8 |
